# Substance misuse and sexual function in adolescents with chronic diseases

**DOI:** 10.1016/j.rppede.2015.10.008

**Published:** 2016

**Authors:** Priscila Araújo, Márcio Guilherme Nunes Carvalho, Marlon van Weelden, Benito Lourenço, Lígia Bruni Queiroz, Clovis Artur Silva

**Affiliations:** a Universidade Católica de Campinas, Campinas, SP, Brazil; b Faculdade de Medicina, Universidade de São Paulo (USP), São Paulo, SP, Brazil; c Medical Faculty, Amsterdam, Netherlands

**Keywords:** Alcoholism, Tobacco, Illicit drugs, Bullying, Adolescent, Chronic disease

## Abstract

**Objective:**

To evaluate alcohol/tobacco and/or illicit drug misuse in Chronic Diseases (CDs).

**Methods:**

A cross-sectional study with 220 CDs adolescents and 110 healthy controls including: demographic/anthropometric data; puberty markers; modified questionnaire evaluating sexual function, alcohol/smoking/illicit drug misuse and bullying; and the physician-conducted CRAFFT (car/relax/alone/forget/friends/trouble) screen tool for substance abuse/dependence high risk.

**Results:**

The frequencies of alcohol/tobacco and/or illicit drug use were similar in both groups (30% vs. 34%, *p*=0.529), likewise the frequencies of bullying (42% vs. 41%, *p*=0.905). Further analysis solely in CDs patients that used alcohol/tobacco/illicit drug versus those that did not use showed that the median current age [15 (11–18) vs. 14 (10–18) years, *p* <0.0001] and education years [9 (5–14) vs. 8 (3–12) years, *p* <0.0001] were significant higher in substance use group. The frequencies of Tanner 5 (*p* <0.0001), menarche (*p* <0.0001) and spermarche (*p*=0.001) were also significantly higher in patients with CDs that used alcohol/tobacco/illicit, likewise sexual activity (23% vs. 3%, *p* <0.0001). A trend of a low frequency of drug therapy was observed in patients that used substances (70% vs. 82%, *p*=0.051). A positive correlation was observed between CRAFFT score and current age in CD patients (*p*=0.005, r=+0.189) and controls (*p*=0.018, r=+0.226).

**Conclusions:**

A later age was evidenced in CDs patients that reported licit/ilicit drug misuse. In CDs adolescent, substance use was more likely to have sexual intercourse. Our study reinforces that these patients should be systematically screened by pediatricians for drug related health behavioral patterns.

## Resumo

**Objetivo:**

Avaliar o uso indevido de álcool/tabaco e/ou de drogas ilícitas em doenças crônicas (DCs).

**Métodos:**

Estudo transversal com 220 adolescentes com DCs e 110 controles saudáveis, incluindo: dados demográficos/antropométricos; marcadores de puberdade; questionário modificado de avaliação da função sexual, abuso de álcool/tabagismo/drogas ilícitas e assédio moral; e o uso do instrumento CRAFFT (car/relax/alone/forget/friends/trouble) pelo médico para o abuso/dependência de substâncias de alto risco.

**Resultados:**

As frequências de uso de álcool/tabaco e/ou drogas ilícitas foram semelhantes em ambos os grupos (30% *vs*. 34%, *p*=0,529), assim como as frequências de assédio moral (42% *vs*. 41%, *p*=0,905). Uma análise mais aprofundada apenas em pacientes com DCs que usaram álcool/tabaco/droga ilícita *versus* aqueles que não usaram mostrou que a idade mediana atual [15 (11-18) *vs*. 14 (10-18) anos, *p* <0,0001] e os anos de escolaridade [9 (5-14) *vs*. 8 (3-12) anos, *p* <0,0001] foram significativamente maiores no grupo que fazia uso das substâncias. As frequências de Tanner 5 (*p* <0,0001), menarca (*p* <0,0001) e espermarca (*p*=0,001) também foram significativamente maiores em pacientes com DCs que usaram álcool/tabaco/drogas ilícitas, assim como a atividade sexual (23% *vs*. 3%, *p* <0,0001). A tendência de baixa frequência de terapia com medicamentos foi observada em pacientes que usaram substâncias (70% *vs*. 82%, *p*=0,051). Observou-se uma correlação positiva entre o score no CRAFFT e a idade atual em pacientes com DCs (*p*=0,005, r=+0,189) e controles (*p*=0,018, r=+0,226).

**Conclusões:**

A idade mais avançada foi demonstrada em pacientes com DCs que relataram uso indevido de drogas lícitas/ilícitas. Em adolescente com DCs, o uso das substâncias resultou em maior propensão à prática de relações sexuais. Nosso estudo reforça que esses pacientes devem ser sistematicamente avaliados pelos pediatras em relação a padrões de comportamento de saúde relacionados com drogas.

## Introduction

Adolescence is a developmental stage characterized by biological and social changes.^1^^,^ 2 Risk patterns may begin in this period of life, including substance misuse (alcohol, tobacco and illicit drugs)^1^ and high-risk sexual behaviors.^3^ Harmful phenomena of peer victimization, such as bullying, is also a relevant problem in healthy adolescents^4^ and in those with chronic diseases (CDs).^5^

Of note, the prevalence of chronic conditions has increased in pediatric population,^6^ and 10% of adolescents suffer from CDs.^7^ However, to our knowledge, misuse of substances evaluated by a screening tool,^8^ as well as assessment of bullying and sexual function, have not been simultaneously studied in an adolescent CDs population.

Therefore, the objectives of the present study were to evaluate alcohol, tobacco and/or illicit drug misuse in adolescent CDs patients and healthy controls. The possible associations between the substance misuse in CDs patients according to sexual function, bullying, demographic data, puberty markers, CDs groups and drug therapy use were also assessed.

## Method

From February to December 2014, a cross-sectional study was carried out involving 220 consecutive outpatient adolescents (current age 10-19 years according to World Health Organization criteria) with pediatric CDs. All of them were followed at the Adolescent Unit of our University Hospital and none of them had unwillingness to participate. The control group included 110 healthy adolescents (rate 2:1), without CDs, consecutively admitted to the outpatient clinic and referred from primary and secondary health care services to the Adolescent Unit of our University Hospital. This study was approved by the Local Ethics Committee of Hospital. All adolescents and their parents signed a consent/assent term.

This study included a modified questionnaire evaluating sexual function,^9^ alcohol/tobacco/illicit drug use and bullying was applied. The Portuguese CRAFFT (mnemonic acronym of car, relax, alone, forget, friends and trouble) screen (CRAFFT/CEASER) version was performed in both groups.^3^^,^ 8 Demographic/anthropometric data and puberty markers assessments were also evaluated.

A pilot study was carried out in 30 consecutive healthy and adolescents with CDs, who were tested and retested 1-2 months. The pre-test evaluated the subject comprehension of the questions, the consistency and coherence of the answers and the time taken to answer the questionnaire. The modified questionnaire included 14 questions with the option of answer "yes/no" or age/number of times about sexual function,^9^ bullying and alcohol/tobacco/illicit drugs use. Sexual function assessment included^9^: age at first sexual intercourse, sexual intercourse in the last month, use of male contraceptive (condom) in the first sexual activity, current use of oral and emergency contraceptive, knowledge of sexual activity by parents and total number of sexual partners. Alcohol/tobacco and drugs [illicit inhalants drug (glue sniffing, aerosol and solvents) and illicit drugs [marijuana, stimulants (cocaine, crack and speed), poppers, LSD, opiates, heroin, crystal meth and ecstasy] use were also assessed: age at alcohol initiation, number of days of alcohol use in the last 30 days, age at smoking initiation, number of days smoking cigarettes in the last 30 days, age at illicit drug initiation and number of days using illicit drugs in the last 30 days. Bullying, which is defined as a recurrent exposure to emotional and/or physical aggression, was obtained by a "yes/no" answer to the question ("Have you ever suffered bullying?"). The questionnaire was strictly confidential and was performed in the absence of legal guardians, relatives and friends.

The Portuguese version of physician-conducted CRAFFT (CRAFFT/CEASER) screen was used and consisted of 9 questions developed to screen adolescents for high-risk alcohol and drug misuse.^8^ This questionnaire is divided in two parts. Part A includes three questions regarding the use of alcohol, marijuana/hashish or another substance in the last twelve months. If the adolescent responded "no" to all three questions, only the question related to "car" of the B-part should be asked. If the adolescent answered "yes" to one of the opening questions, all of the questions of part B should be asked. B-part contained six questions, which are signs of problematic substance use. One point was given to each "yes" answer in the B-part of the questionnaire. The score ranged from 0 to 6. A total score of ≥2 indicated high risk for substance abuse/dependence and a need for additional assessment.^3^^,^ 8

Regarding socio-demographic and anthropometric data, current age, gender, years of education, weight and height were evaluated. Body mass index (BMI) was defined by the formula: weight in kilograms/height in square meters. The Brazilian socio-economic status were classified according to the ABEP (Associação Brasileira de Empresas de Pesquisa).^10^

Secondary sexual characteristics were classified according to Tanner pubertal changes.^11^ Age at first menstruation (menarche) and age at first ejaculation (spermarche) were registered based on memory recollection.

Chronic diseases, with disease duration more than three months, were classified in: Allergic/Immunologic, Cardiopulmonary, Dermatologic, Endocrinologic, Gastroenterologic, Hematologic, Neuropsychiatric, Renal and Rheumatic conditions. The use of drug therapy was also assessed.

The test-retest reliability of the modified questionnaire was verified for CDs population and healthy controls using the Kappa index. Results were presented as the mean ± standard deviation (SD) or median (range) for continuous and number (%) for categorical variables. Data were compared by *t* or Mann-Whitney tests in continuous variables to evaluate differences between adolescents with CDs and controls, and between subgroups. For categorical variables, differences were assessed by Fisher's exact or Pearson chi-square tests. Spearman rank correlation coefficient was used for correlations between CRAFFT score and age. The level of significance was set at 5% (*p* <0.05).

## Results

The kappa index for test-retest was 0.850, demonstrating excellent reliability for the adolescents' responses.

The frequencies of alcohol, tobacco and/or illicit drug use were similar in both adolescents with CDs and healthy controls (30% vs. 34%, *p*=0.529). The frequency of alcohol consumption in the past 30 days was significantly lower in adolescents with CDs compared with controls (6% vs. 27%, *p*=0.005). Illicit drugs were used by three CDs patients (marijuana, cocaine and/or poppers) and three controls (marijuana) (*p*=0.404). No difference was observed in the frequency of smoking (*p*=0.114). CRAFFT score ≥2 was similar in both groups (4% vs. 5%, *p*=0.575). The frequency of bullying was alike in both groups (36% vs. 39%, *p*=0.905) (Table 1). The median of age onset smoking was 14 years (11-15) in adolescents with CDs and was 14 years (7-15) in healthy controls. The median of age onset ilicit drug use was 14.5 years (14-15) in adolescents with CDs and was 14 years in healthy controls.

**Table 1:** Alcohol, smoking and illicit drug use, and bullying in adolescents with chronic diseases and healthy controls.

Variables	Chronic diseases (n=220)	Healthy controls (n=110)	*p*-value
**Alcohol, smoking and/or illicit drug use**	66 (30)	37 (34)	0.529
Alcohol use	66 (30)	37 (34)	0.529
Age at onset alcohol, yrs	14 (6-17)	14 (7-18)	0.935
Drinking alcohol in past 30 days	4/66 (6)	10/37 (27)	**0.005**
Smoking use	5 (2)	7 (6)	0.114
Illicit drug use	3 (1)	3 (3)	0.404
CRAFFT score in the last 12 months (0-6)	0 (0-5)	0 (0-5)	0.712
CRAFFT score ≥2	9 (4)	6 (5)	0.575

*CRAFFT item*			
Car	41 (19)	17 (15)	0.474
Relax	6/36 (17)	5/27 (19)	1.000
Alone	1/36 (3)	2/27 (7)	0.572
Forget	1/36 (3)	4/27 (15)	0.155
Familiy/friends	8/36 (22)	7/27 (26)	0.733
Trouble	6/36 (17)	0/27 (0)	**0.033**
**Bullying**	80 (36)	43 (39)	0.905

*Sexual function*			
Sexual activity	20 (9)	16/109 (15)	0.126
First sexual activity age, yrs	15 (8-16)	14 (10-17)	0.859
Sexual intercourse in last month	3/20 (15)	6/16 (38)	0.146
Condom at the first sexual activity	19/20 (95)	13/16 (81)	0.303
N Oral contraception use in females	2/13 (15)	3/13 (23)	1.000
Emergency contraceptive use^a^	1/13 (8)	6/13 (46)	0.073
Knowledge of sexual activity by parents	9/20 (45)	11/16 (69)	0.154
Total number of sexual partner, number	1 (1-16)	1 (1-5)	0.586

The results are presented in n (%) and median (range), CRAFFT (car, relax, alone, forget, friends, trouble) screening test; BMI, body mass index.

a One healthy control used oral contraception before sexual activity.

A positive correlation was evidenced between CRAFFT score and current age in CDs patients (*p*=0.005, r=+0.189) and healthy controls (*p*=0.018, r=+0.226), likewise between CRAFFT score and education years in CDs (*p*=0.015, r=+0.164) and controls (*p*=0.039, r=+0.197). A positive correlation was observed between CRAFFT score and age of alcohol onset in CDs patients (*p*=0.021, r=+0.286) (Table 1).

The median current age was similar between adolescent with CDs and healthy controls [14 (10-18) vs. 14 (10-18) years, *p*=0.778], likewise the frequency of female gender (65% vs. 67%, *p*=0.742), years of education [9 (3-14) vs. 8 (4-12) years, *p*=0.857], Brazilian socio-economic classes B or C (95% vs. 94%, *p*=0.365) and between Tanner 3, 4 and 5 (*p*=0.582). The median menarche age was significantly higher in the former group [12 (8-16) vs. 11 (9-15) years, *p*=0.009]. No difference was evidenced in the age at spermarche in both groups (*p*=0.642) (Table 2).

**Table 2:** Demographic data and puberty markers in adolescents with chronic diseases and healthy controls.

Variables	Chronic diseases (n=220)	Healthy controls (n=110)	*p*-value
*Demographic data*			
Current age, yrs	14 (10-18)	14 (10-18)	0.778
Female gender	144 (65)	74 (67)	0.742
BMI, kg/m^2^	21 (12-39)	21 (13-29)	0.091
Social economic class B and C	211 (95)	103 (94)	0.365
Education, yrs	9 (3-14)	8 (4-12)	0.857

*Puberty markers*			
Tanner 3	53 (24)	23 (21)	-
Tanner 4	52 (24)	29 (26)	-
Tanner 5	77 (35)	46 (42)	0.582
Menarche	113/144 (78)	65/74 (88)	0.091
Menarche age, yrs	12 (8-16)	11 (9-15)	**0.009**
Spermarche	38/76 (50)	23/36 (64)	0.168
Spermarche age, yrs	12 (10-15)	13 (8-14)	0.642

The results are presented in n (%) and median (range); BMI, body mass index.

Further analysis solely of patients with CDs regarding alcohol/tobacco/illicit drug misuse showed that the median current age [15 (11-18) vs. 14 (10-18) years, *p* <0.0001] and education years [9 (5-14) vs. 8 (3-12) years, *p* <0.0001] were significant higher in those that used the aforementioned substances. The frequencies of Tanner 5 (*p* <0.0001), menarche (*p* <0.0001) and spermarche (*p*=0.001) were also significantly higher in the former group, likewise sexual activity (23% vs. 3%, *p* <0.0001) (Table 3).

**Table 3:** Demographic data, puberty markers and bullying in adolescents with chronic diseases according to alcohol, smoking and illicit drug use.

Variables	Use alcohol, smoking and/or illicit drug (n=66)	Non use alcohol, smoking and/or illicit drug (n=154)	*p*-value
*Demographic data*			
Current age, yrs	15 (11-18)	14 (10-18)	< **0.0001**
Female gender	43 (65)	101 (66)	0.951
BMI, kg/m^2^	21 (17-38)	21 (12-39)	0.275
Social economic class B and C	62 (94)	149 (97)	0.457
Education, yrs	9 (5-14)	8 (3-12)	< **0.0001**

*Puberty markers*			
Tanner 3	13 (20)	40 (26)	-
Tanner 4	15 (23)	37 (24)	-
Tanner 5	36 (55)	41 (27)	< **0.0001**
Menarche	42/43 (98)	71/101 (70)	< **0.0001**
Menarche age, yrs	12 (9-16)	12 (8-15)	0.348
Spermarche	18/23 (78)	20/53 (38)	**0.001**
Spermarche age, yrs	12 (10-14)	12.50 (10-15)	0.668

*Sexual function*			
Sexual activity	15 (23)	5 (3)	< **0.0001**
First sexual activity age, yrs	14.50 (8-16)	15 (14-15)	0.702
Sexual intercourse in last month	3/15 (20)	0/5 (0)	0.539
Condom at the first sexual activity	14/15 (93)	5/5 (100)	1.000
N Oral contraception use in females	2/11 (18)	0/2(0)	1.000
Emergency contraceptive use	1/11 (9)	0/2 (0)	1.000
Knowledge of sexual activity by parents	6/15 (40)	3/5 (60)	0.617
Total number of sexual partner, number	1 (1-6)	1 (1-16)	1.000
**Bullying**	24 (36)	55 (36)	0.685

The results are presented in n (%) and median (range); CRAFFT (car, relax, alone, forget, friends, trouble) screening test; BMI, body mass index.

Of our 9 patients with CDs with CRAFFT score ≥2, rheumatic diseases were found in 8 (juvenile idiopathic arthritis in three patients, juvenile dermatomyositis in one, childhood-onset systemic lupus erythematosus in one, Sjögren syndrome in one, Takayasu arteritis in one and Henoch-Schönlein purpura in one) and Allergic disease in one patient (asthma).

The frequencies of chronic conditions in patients that used alcohol/tobacco/illicit drug versus those that did not use were similar (*p*>0.05). A trend of a low frequency of drug therapy was evidenced in patients that used substances compared to those that did not used (70% vs. 82%, *p*=0.051) (Table 4).

**Table 4:** Adolescent chronic diseases and drug therapy according to alcohol, smoking and illicit drug use the results are presented in n (%).

Variables	Use alcohol, smoking and/or illicit drug (n=66)	Non use alcohol, smoking and/or illicit drug (n=154)	*p*-value
*Adolescent chronic diseases*			
Immunologic	3 (5)	2 (1)	0.160
Dermatologic	3 (5)	3 (2)	0.367
Endocrinologic	12 (18)	33 (21)	0.715
Gastroenterologic	2 (3)	11 (7)	0.352
Neuropsychiatric	7 (11)	20 (13)	0.822
Allergic/cardiopulmonary	9 (14)	23 (15)	1.000
Renal	5 (8)	5 (3)	0.170
Rheumatic	46 (70)	112 (73)	0.743
Hematologic	0 (0)	1 (1)	1.000
*Drug therapy for chronic diseases*	46 (70)	126 (82)	**0.051**

## Discussion

Our study evaluated simultaneously adolescent's health problems in CDs patients and healthy controls. In adolescents with CDs, substance use was observed in later age and was more likely to sexual intercourse compared to those that did not use licit/illicit drug.

The advantage of this study was the assessment of physician-conducted CRAFFT (CEASER) screening tool for substance misuse in adolescents.^3^^,^ 8 The use of the questionnaire with excellent test-retest reliability regarding sexual function, bullying and substances use was also relevant. Additionally, a healthy control group with comparable age, gender, education and socio-economic class was also observed in our study, since these data were previously related with bullying and substance misuse.^1^^,^ 3 ^,^ 12 ^,^ 13

Alcohol intake was reported by approximately one third of our CDs patients, even though a low intake frequency in the last month. The prevalence of alcohol use in adolescents varies from 7.8% to 78.9%^12^^,^ 14 ^,^ 15 around the world and from 26% to 71% in Brazil.^16^^-^ 21 There are studies in adolescents with specific CDs, generally with small samples and variable frequencies of alcohol use (4.8%-70%), such as juvenile idiopathic arthritis,^22^ obesity and overweight,^23^ asthma,^24^ cystic fibrosis and sickle cell disease.^25^ This finding in our CDs patients may be related to the availability of alcohol at parties, pubs, stores and homes in Brazil.^16^

Importantly in the evaluation of only adolescents with CDs, we observed that use of alcohol, tobacco and illicit drugs occurred in CDs patients that presented higher age, higher education background and post pubertal markers. The avoidance of substance use in early adolescence may be related to overprotection of parents and primary caregivers. In contrast, a more liberal parental attitude may contribute to an increase in substance misuse later on in life. Moreover, severe CDs that required higher frequencies of medication may have contributed to the low use of illicit and licit drugs.

The adolescents may develop a drinking pattern that is socially acceptable and a small group of CDs patients' misused alcohol more frequently. We applied a screening procedure for substance misuse and 4% of our CDs patients had a higher risk for substance abuse/dependence. A positive correlation was also evidenced between CRAFFT score and current age, indicating a higher risk for harmful substance misuse in older CDs adolescents.

Smoking was reported only by 2% of our CDs patients. This aspect has also been observed in adolescents of our continental country due to ban on smoking advertising,^26^ use of warnings on cigarettes packages^27^ and prohibition in public places.^28^ The use of any illicit drug (marijuana, solvents, cocaine or crack) was reported in 2.8% of adolescents in a Brazilian survey,^21^ and this result was higher than in our CDs patients.

Interestingly, CDs substance users engaged more in sexual activity, and may present a higher risk for sexually transmitted diseases, unwanted pregnancy and low contraceptive use.^29^^,^ 30 This finding may also be related to the fact that the patients were older and probably had higher sexual maturity. However, this study did not evaluate if drinking influenced high-risk sexual behavior in CDs patients.

In addition, bullying was frequently reported in CDs and seems not to be linked to substance use. Adolescents with CDs may suffer from this victimization,^4^^,^ 5 may induce anxiety-depressive disorder and interfere with proper adherence to medication use. A prospective study evaluating prevalence and intensity of bullying in a large sample of CDs patients will be required.

The main limitation of the present study was the use of a convenience sample for CDs patients in a tertiary University Hospital, predominantly of rheumatic, endocrinologic, allergic and cardiopulmonary diseases. A small sample may also have reduced our power to detect differences between the two groups and the cross sectional design nature of our study did not allow the evaluation of temporality between exposure and outcome. We did not evaluate disease activity criteria for all CDs, and indeed this finding may modify the behavior. Moreover, the healthy controls and CDs patients were referred to the adolescent clinic of our University Hospital to guidance about sexual function and birth control, emotional problems and drugs issues.

A need for additional assessment and therapeutic intervention with a multidisciplinary and multiprofessional team is required for CDs adolescents and families at risk for substance misuse. Confidential discussion of illicit and licit drugs and other potentially risky behaviors should be included in the routine care of clinics that followed-up CDs patients.

In conclusion, a later age was evidenced in CDs patients that reported licit/ilicit drug use. CDs substance users were more likely to have sexual intercourse. Our study reinforces that these patients should be systematically screened by pediatricians for sexual, alcohol and drug related health behavioral patterns.

## References

[1] Sawyer SM, Afifi RA, Bearinger LH, Blakemore SJ, Dick B, Ezeh AC (2012). Adolescence: a foundation for future health. Lancet.

[2] Jannini SN, Dória-Filho U, Damiani D, Silva CA (2011). Musculoskeletal pain in obese adolescents. J Pediatr (Rio J).

[3] Levy S, Sherritt L, Gabrielli J, Shrier LA, Knight JR (2009). Screening adolescents for substance use-related high-risk sexual behaviors. J Adolesc Health.

[4] Sentenac M, Gavin A, Arnaud C, Molcho M, Godeau E, Nic Gabhainn S (2011). Victims of bullying among students with a disability or chronic illness and their peers: a cross-national study between Ireland and France. J Adolesc Health.

[5] Pittet I, Berchtold A, Akré C, Michaud PA, Surís JC (2010). Are adolescents with chronic conditions particularly at risk for bullying?. Arch Dis Child.

[6] Ng M, Fleming T, Robinson M, Thomson B, Graetz N, Margono C (2014). Global, regional, and national prevalence of overweight and obesity in children and adults during 1980-2013: a systematic analysis for the Global Burden of Disease Study 2013. Lancet.

[7] Suris JC, Michaud PA, Viner R (2004). The adolescent with a chronic condition. Part I: Developmental issues. Arch Dis Child.

[8] Knight JR, Schram P Portuguese version of CRAFFT screen (CEASER).

[9] Febronio MV, Pereira RM, Bonfa E, Takiuti AD, Pereyra EA, Silva CA (2007). Inflammatory cervicovaginal cytology is associated with disease activity in juvenile systemic lupus erythematosus. Lupus.

[10] ABEP.

[11] Marshall JC, Tanner JM (1970). Variations in patterns of pubertal changes in boys and girls. Arch Dis Child.

[12] Atilola O, Stevanovic D, Balhara YP, Avicenna M, Kandemir H, Knez R (2014). Role of personal and family factors in alcohol and substance use among adolescents: an international study with focus on developing countries. J Psychiatr Ment Health Nurs.

[13] Moreira TC, Belmonte EL, Vieira FR, Noto AR, Ferigolo M, Barros HM (2008). Community violence and alcohol abuse among adolescents: a sex comparison. J Pediatr (Rio J).

[14] Seil KS, Desai MM, Smith MV (2014). Sexual orientation, adult connectedness, substance use, and mental health outcomes among adolescents: findings from the 2009 New York City Youth Risk Behavior Survey. Am J Public Health.

[15] Poelen EA, Scholte RH, Engels RC, Boomsma DI, Willemsen G (2005). Prevalence and trends of alcohol use and misuse among adolescents and young adults in the Netherlands from 1993 to 2000. Drug Alcohol Depend.

[16] Malta DC, Machado IE, Porto DL, da Silva MM, de Freitas PC, da Costa AW (2014). Alcohol consumption among Brazilian Adolescents according to the National Adolescent School-based Health Survey (PeNSE 2012). Rev Bras Epidemiol.

[17] Malta DC, Bernal RT (2014). Comparison of risk and protective factors for chronic diseases in the population with and without health insurance in the Brazilian capitals, 2011. Rev Bras Epidemiol.

[18] Barbosa VC, Campos W, Lopes Ada S (2012). Prevalence of alcohol and tobacco use among Brazilian adolescents: a systematic review. Rev Saude Publica.

[19] Duailibi LB, Ribeiro M, Laranjeira R (2008). Profile of cocaine and crack users in Brazil. Cad Saude Publica.

[20] Malta DC, Mascarenhas MD, Porto DL, Duarte EA, Sardinha LM, Barreto SM (2011). Prevalence of alcohol and drug consumption among adolescents: data analysis of the National Survey of School Health. Rev Bras Epidemiol.

[21] Madruga CS, Laranjeira R, Caetano R, Pinsky I, Zaleski M, Ferri CP (2012). Use of licit and illicit substances among adolescents in Brazil - a national survey. Addict Behav.

[22] Nash AA, Britto MT, Lovell DJ, Passo MH, Rosenthal SL (1998). Substance use among adolescents with juvenile rheumatoid arthritis. Arthritis Care Res.

[23] Terres NG, Pinheiro RT, Horta BL, Pinheiro KA, Horta LL (2006). Prevalence and factors associated to overweight and obesity in adolescents. Rev Saude Publica.

[24] Kim O, Kim BH (2013). Association of asthma symptoms with cigarette smoking and alcohol consumption in Korean adolescents. Nurs Health Sci.

[25] Britto MT, Garrett JM, Dugliss MA, Daeschner CW, Johnson CA, Leigh MW (1998). Risky behavior in teens with cystic fibrosis or sickle cell disease: a multicenter study. Pediatrics.

[26] Galduróz JC, Fonseca AM, Noto AR, Carlini EA (2007). Decrease in tobacco use among Brazilian students: a possible consequence of the ban on cigarette advertising?. Addict Behav.

[27] Nascimento BE, Oliveira L, Vieira AS, Joffily M, Gleiser S, Pereira MG (2008). Avoidance of smoking: the impact of warning labels in Brazil. Tob Control.

[28] Silva ST, Martins MC, Faria FR, Cotta RM (2014). Combating smoking in Brazil: the strategic importance of government actions. Cien Saude Colet.

[29] Araujo DB, Borba EF, Abdo CH, Souza Lde A, Goldenstein-Schainberg C, Chahade WH (2010). Sexual function in rheumatic diseases. Acta Reumatol Port.

[30] Silva CA, Hilario MO, Febronio MV, Oliveira SK, Almeida RG, Fonseca AR (2008). Pregnancy outcome in juvenile systemic lupus erythematosus: a Brazilian multicenter cohort study. J Rheumatol.

